# Effects of Qidantang Granule on early stage of diabetic kidney disease in rats

**DOI:** 10.18632/aging.204121

**Published:** 2022-06-13

**Authors:** Tengfei Wu, Xinyu Yang, Yilei Cong, Shisi Xia, Bowen Liu, Ran Zou, Juanhua Zeng, Hua Yang

**Affiliations:** 1 Department of Endocrinology, Longhua Hospital, Shanghai University of TCM, Shanghai 200032, China

**Keywords:** PI3K, OS, inflammation, DKD, TCM

## Abstract

Diabetic kidney disease (DKD), is one of the most common vascular diseases caused by diabetes, eventually progressing into glomerular sclerosis. Qidantang Granule is a traditional Chinese medicine that is commonly used for DKD. However, there is still no experimental evidence for its effectiveness on DKD. 8-week-old Sprague Dawley male rats were fed on high-fat and high-sugar diet for 4 weeks, and then intraperitoneally injected with 35 mg/kg streptozotocin (STZ) to induce diabetes. Diabetic rats were randomly divided into three groups, and orally administrated with vehicle, 50 mg/kg or 200 mg/kg Qidantang Granule respectively, once daily for 9 weeks. Qidantang Granule effectively reduced food and water intake, body weight and fasting blood glucose, decreased inflammation and oxidative stress, ameliorated renal injury through suppressing PI3K signaling pathway in STZ-induced DKD rats. Our results provide experimental evidence to demonstrate the pharmacological mechanism of Qidantang Granule in the treatment of DKD.

## INTRODUCTION

Diabetic kidney disease (DKD), is one of the most common vascular diseases caused by diabetes, eventually progressing into glomerular sclerosis [1, 2]. DKD is characterized by diffuse thickening of the glomerular basement membrane, and morphological changes such as mesangial matrix proliferation and expansion, leading to renal insufficiency [3, 4]. Without proper and timely treatment, DKD will eventually develop into end-stage renal disease (ESRD). Moreover, DKD is also a major cause of mortality and morbidity in diabetic patients all over the world [5]. According to the latest statistics from World Health Organization, about 40% of diabetic patients will suffer from DKD [6]. The common treatment of DKD includes intensive hypoglycemic and application of angiotensin-converting enzyme inhibitor (ACEI), angiotensin-receptor blockers (ARB) or other drugs to control hypertension, or lower lipid. However, the outcome of these strategies is unsatisfied. Therefore, new candidate drugs for treating DKD need to be developed and explored to attenuate the progression of DKD.

Previous publications have shown that persistent hyperglycemia of patients with diabetes altered hemodynamics and induced oxidative stress in the peripheral blood, therefore leading to severe damage to local renal cells, including podocytes and glomerular capillary endothelial cells [7]. Among these cells, podocytes are terminally differentiated cells, which play an important role in the glomerulus [8, 9]. Due to terminal differentiation, podocytes can’t regenerate once damaged. Hyperglycemia causes hypertrophy and detachment of podocytes. Therefore, the amounts of epithelia cells in urine as the biomarker of early stage of DKD is a better biomarker than albumin amounts in the urine [10]. Moreover, avoiding podocyte damage was believed as the key to prevent the occurrence of DKD [11].

Traditional Chinese Medicine (TCM) has been applied to treat DKD in clinical practice for a long time, and it has been approved to effectively attenuate symptoms of DKD, reduce proteinuria, and delay the progression of end-stage uremia [12, 13]. Qidantang Granule is a common TCM to treat early stage of DKD. Our group has been worked on the clinical and experimental research of Qidantang Granule in the treatment of early stage of DKD for almost 20 years, and establishes a relatively mature therapeutic plan, which was supported by the results of clinical trial (ChiCTR220005601). However, its detailed mechanisms remain largely unknown. Therefore, our current study aimed to explore the protective effect of Qidantang Granule on podocytes in DKD rats, to provide theoretical and experimental evidence of Qidantang Granules in the treatment of early DKD.

## MATERIALS AND METHODS

### DKD rat model

DKD rat model was performed as previously described [14]. Briefly, 8-week-old Sprague Dawley male rats were fed on high-fat and high-sugar diet for 4 weeks, and then intraperitoneally injected with 35 mg/kg streptozotocin (STZ). After 3 days, rats with >13.9 mmol/L fasting blood glucose and >16.7 mmol/L blood glucose were selected as diabetic rats. Diabetic rats were randomly divided into three groups, and orally administrated with vehicle, low-dose (50 mg/kg) or high-dose (200 mg/kg) Qidantang Granule respectively, once daily for 9 weeks after the success establishment of DKD model. Fasting blood glucose, body weight, food and water intake were monitored every week for 9 weeks. Then 24 h urine, blood samples and kidney tissues were collected. Blood glucose was measured using a glucometer (Accu-Check Active, Roche). All the animal experiments were approved by the ethics committee of Longhua Hospital, Shanghai University of TCM.

### Urine parameters’ analysis

The rats were placed in the promethion cages individually and 24 h urine was collected. 3 mL of urine was centrifuged at 3,000 rpm for 10 min at 4° C, then supernatant was collected and analyzed by an automatic biochemical analyzer (Olympus, Japan) to determine the 24 h microalbumin (mALB) in urine and albumin/creatinine ratio (ACR).

### Blood parameters’ analysis

Blood samples were centrifuged at 6,000 rpm for 15 min to get serum. Total cholesterol (TC), triacylglycerol (TG), blood urea nitrogen (BUN), and the serum creatinine (SCr) were determined using the automatic biochemical analyzer.

Interleukin-6 (IL-6) (#R6000B), interleukin-1β (IL-1β) (#RLB00) and tumor necrosis factor-α (TNF-α) (#RTA00) levels in the serum were measured using commercial ELISA kits (R&D Systems, Minneapolis, MN, USA) according to the manufacture’s instruction.

### Oxidative stress analysis

Renal tissue from each group was ground and centrifuged at 9,000 rpm for 10 min at 4° C to get supernatant. The activities of malondialdehyde (MDA) (#NWK-MDA01 Northwest Life Science, Vancouver, WA, USA), superoxide dismutase (SOD) (#NWK-SOD02, Northwest Life Science), and lactate dehydrogenase (LDH) (#13809, Zhonghao Biotech, Beijing, China) in renal tissues were measured using MDA, LDH, and SOD assay kits respectively in accordance with the experimental instruction.

### qRT-PCR

Total mRNA was extracted from renal tissue using Trizol buffer (Invitrogen, Waltham, MA, USA), then transcripted into cDNA using MLV Reverse Transcriptase. qRT-PCR was performed as previously described [15]. The primers are as follows:

*Nephrin*

F: 5′-GCATAGCCAGAGGTGGAAATCC

R: 5′-GAACGGTCATCACCAGCACACT

*Podocin*

F: 5′-GTGGAAGCTGAGGCACAAAGAC

R: 5′-CAGCGACTGAAGAGTGTGCAAG

*Desmin*

F: 5′-GCGGCTAAGAACATCTCTGAGG

R: 5′-ATCTCGCAGGTGTAGGACTGGA

*GAPDH*

F: 5′-CATCACTGCCACCCAGAAGACTG

R: 5′-ATGCCAGTGAGCTTCCCGTTCAG

### Western blot

The same amounts of renal tissues were homogenized in RIPA buffer and centrifuged at 12,000 rpm for 10 min to get supernatant as total protein. Then Western blot was performed as previously described [16]. The primary antibodies used in this study included p-PI3K (#SAB5500162, Sigma, St. Louis, MO, USA), PI3K (#ab154598, Abcam, Cambridge, MA, USA), p-AKT (#4060, Cell Signaling Technology, Danvers, MA, USA), AKT (#4691, Cell Signaling Technology), p-mTOR (#5536, Cell Signaling Technology), mTOR (#2983, Cell Signaling Technology), β-Actin (#4970, Cell Signaling Technology). β-Actin was employed as a loading control.

### Statistical analysis

Data were expressed as means ± standard deviation (SD), using SPSS 24.9 for one-way analysis of variance (ANOVA) followed with a Tukey post hoc test, two-way ANOVA followed with a Bonferroni post hoc test. P<0.05 was regarded as significant differences.

## RESULTS

### Qidantang Granule reduced diabetes-induced food intake, water intake, body weight and fasting blood glucose in DKD rats

**Figure 1:**
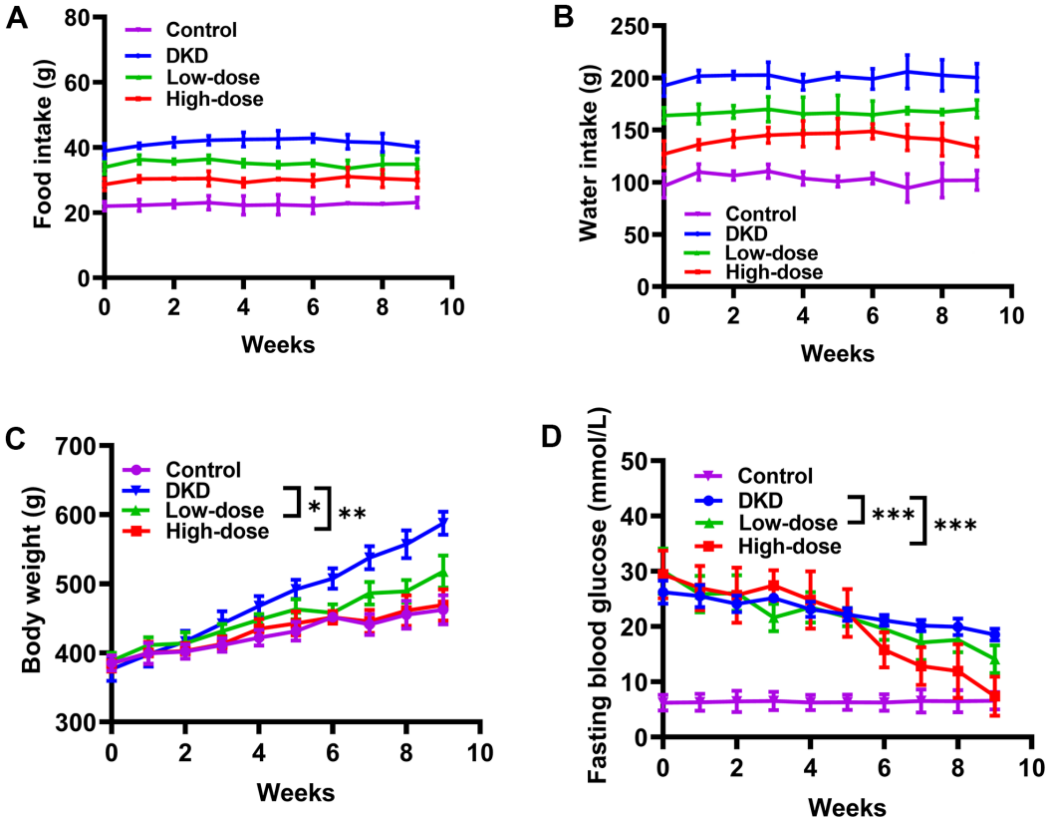
**Effects of Qidantang Granule on DKD rats.** The DKD rats were administered with 50 mg/kg (low-dose) or 200 mg/kg (high-dose) Qidantang Granule for 9 weeks by gavage. (**A**) Food intake of rats in 1-9 weeks. (**B**) Water drink of rats in 1-9 weeks. The weight (**C**) and fasting blood glucose (**D**) of the rats in each group are measured weekly. n=6. *p <0.05, **p < 0.01 and ***p < 0.001 vs DKD group.

To evaluate the effect of Qidantang Granule in DKD rats, we chose two doses (low-dose: 50 mg/kg; high-dose: 200 mg/kg) to gavage DKD rats for 4 weeks. Compared to control mice, STZ-induced DKD mice displayed increased food intake (Figure 1A), water intake (Figure 1B), body weight (Figure 1C) and fasting blood glucose levels (Figure 1D), and all of which could be dose-dependently decreased by Qidantang Granule (Figure 1A–1D). Moreover, Qidantang Granule also time-dependently reduced fasting blood glucose of DKD rats, and high-dose of Qidantang Granule lowered fasting blood glucose to be close to the control group (Figure 1D). In addition, it was found that Qidantang Granule treatment decreased insulin resistance in the DKD rats (Supplementary Figure 1). This suggested that Qidantang Granule had inhibitory effect on blood glucose in DKD rats.

### Qidantang Granule improved biomarkers of kidney function in DKD rats

**Figure 2:**
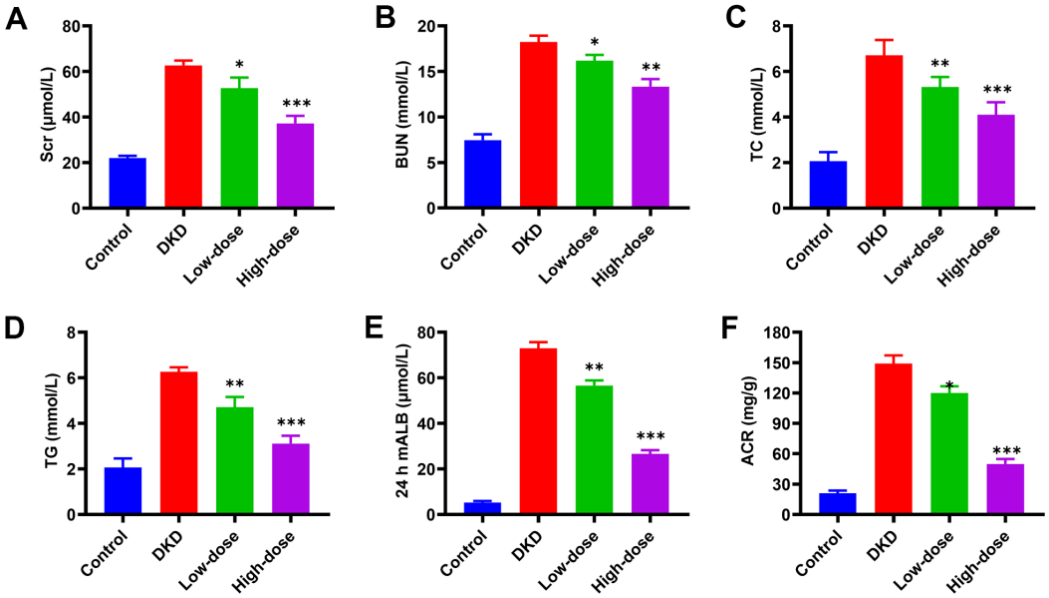
**Effects of Qidantang Granule on serum and urine biochemical indicators in DKD rats.** The DKD rats were administered with 50 mg/kg (low-dose) and 200 mg/kg (high-dose) Qidantang Granule for 9 weeks by gavage. (**A**) SCr levels in the serum of rats in each group; (**B**) BUN levels in the serum of rats in each group; (**C**) TC levels in the serum of rats in each group; (**D**) TG levels in the serum of rats in each group; (**E**) mALB levels in the urine of rats in each group; (**F**) ACR levels in the urine of rats in each group. n=6. *p < 0.05, **p < 0.01 and ***p < 0.001 vs DKD group.

Compared to control group, STZ injection significantly impaired kidney function in rats, evidenced by the increased biomarkers in the serum, including serum creatinine (sCr) (Figure 2A), blood urea nitrogen (BUN) (Figure 2B), total cholesterol (TC) (Figure 2C) and triglyceride (TG) (Figure 2D), and the upregulated biomarkers in the urine, such as albumin-to-creatinine ratio (ACR) (Figure 2E) and 24 h microalbumin (24 h mALB) (Figure 2F). All these biomarkers could be dose-dependently reduced by Qidantang Granule in DKD rats.

### Qidantang Granule attenuated inflammation and oxidative stress in DKD rats

**Figure 3:**
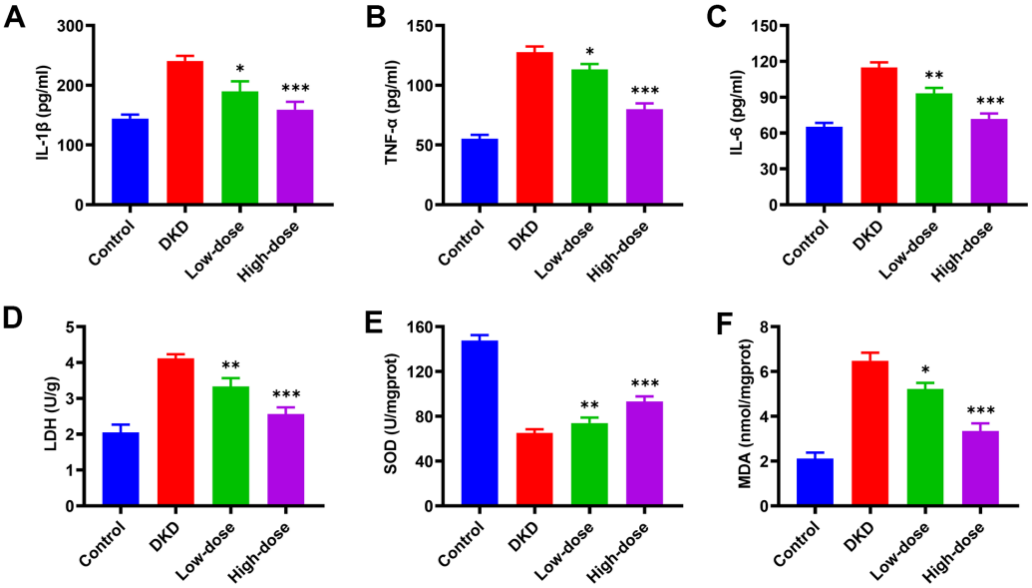
**Effects of Qidantang Granule on serum inflammatory factors and renal oxidative stress factors in DKD rats.** The DKD rats were administered with 50 mg/kg (low dose) and 200 mg/kg (high dose) Qidantang Granule for 9 weeks by gavage. (**A**–**C**) the expression of inflammatory factors TNF-α, IL-1β, and IL-6 in the serum of rats in each group; (**D**–**F**) the activities of LDH, SOD, and MDA in the renal tissue of rats in each group; n=6. *p < 0.05, **p < 0.01 and ***p < 0.001 vs DKD group.

Next, we evaluated the effect of Qidantang Granule on inflammation and oxidative stress in DKD rats. DKD rats displayed increased secretions of inflammatory factors in the serum, including IL-1β (Figure 3A), TNF-α (Figure 3B) and IL-6 (Figure 3C), which indicated the inflammation was activated in DKD rats. Activated inflammation was significantly reduced by Qidantang Granule in a dose-dependent way. Furthermore, compared to control group, STZ injection induced oxidative stress in rats, as evidenced by the increased levels of LDH (Figure 3D) and MDA (Figure 3E) as well as decreased SOD (Figure 3F), and all of which could be dose-dependently restored by Qidantang Granule. These data suggested that Qidantang Granule attenuated inflammation and oxidative stress in DKD rats.

### Qidantang Granule ameliorated kidney injury in DKD rats

**Figure 4:**
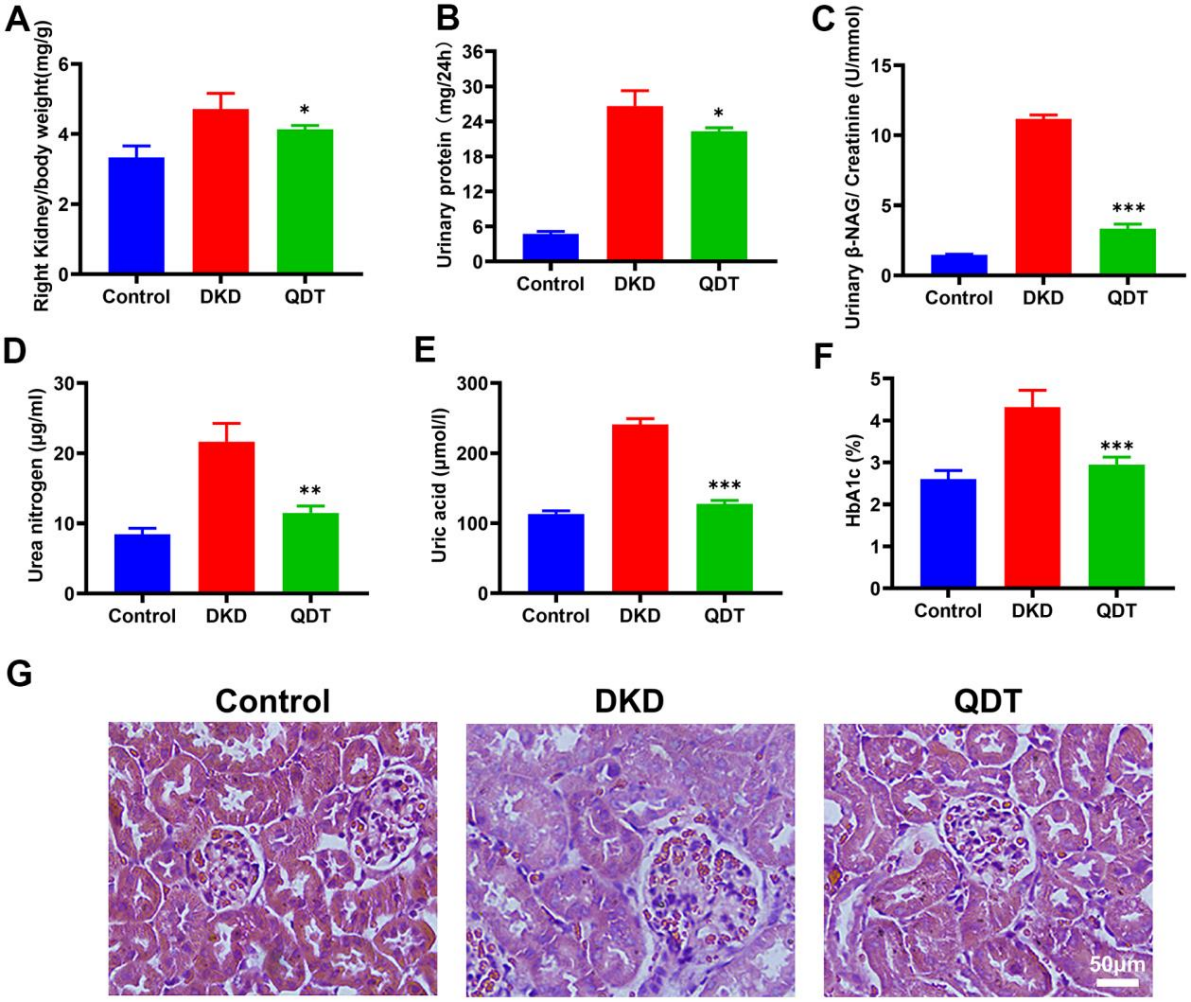
**Qidantang Granule alleviated morphological changes of kidney and improved renal function.** (**A**) Right kidney/body weight; (**B**) Urine protein of 24 h; (**C**) Urinary β-NAG/urinary creatinine; (**D**) Serum urea nitrogen; (**E**) Serum uric acid; (**F**) The percentage of HbA1C. (**G**) H&E staining showed the tissue damage with histology of the kidney. Data are expressed as means SD. n=6. *p < 0.05, **p < 0.01 and ***p < 0.001 vs. DKD group.

Because the high dose (200 mg/kg) of Qidantang granule had a better protective effect on the kidney than the low dose (50 mg/kg), we chose the high dose for the following studies. Compared to control group, DKD rats displayed severe renal injury. All the biomarkers of renal injury in the urine were remarkably upregulated in DKD rats, including right kidney/body weight (Figure 4A), urine protein of 24 h (Figure 4B), urinary β-NAG/urinary creatinine (Figure 4C), serum urea nitrogen (Figure 4D), serum uric acid (Figure 4E), HbAc1 (Figure 4F). In addition, H&E staining showed that Qidantang Granule could attenuate renal damage in DKD rats (Figure 4G). Qidantang Granule significantly reduced all these biomarkers of renal injury, which suggested that Qidantang Granule significantly alleviated renal injury in DKD rats. Moreover, rats with insulin treatment had significant lower serum urea nitrogen, serum uric acid, and serum creatinine than DKD rats (Supplementary Figure 2A–2C), indicating that STZ-induced kidney damage was associated with pancreatic β-cell damage.

### Qidantang Granule ameliorated renal injury by inhibiting PI3K/Akt/mTOR signaling pathway in DKD rats

**Figure 5:**
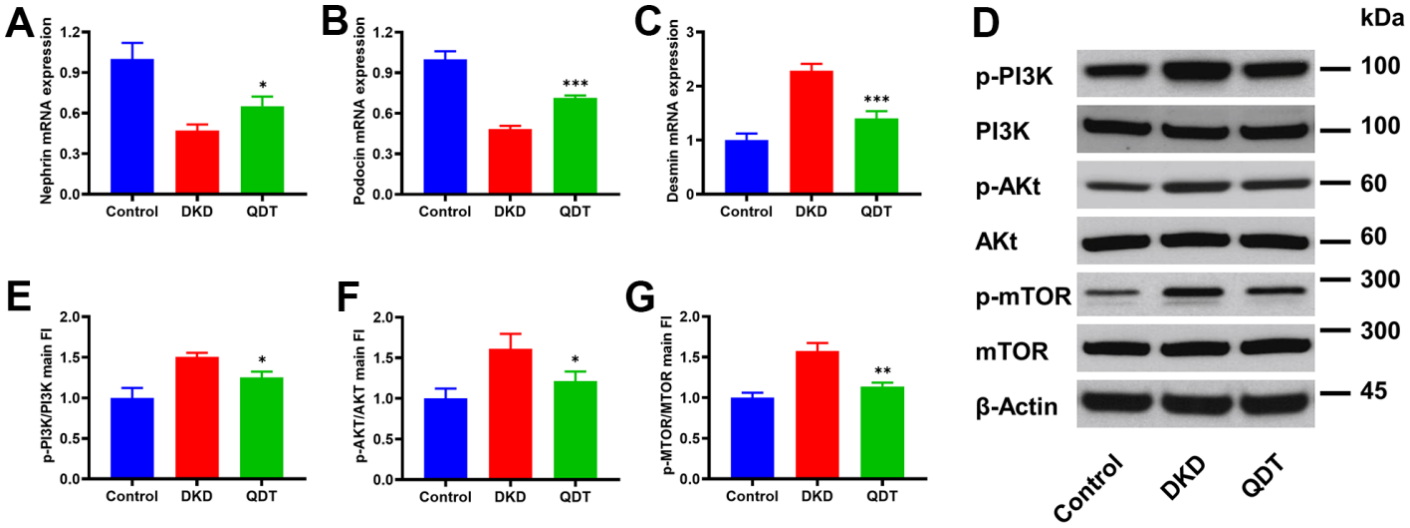
**Qidantang Granule ameliorates renal injury by inhibiting PI3K/Akt/mTOR signaling pathway in DKD rats.** The rats were administrated with 200 mg/kg Qidantang Granule by gavage for 9 weeks. qRT-PCR to detect the mRNA levels of Nephrin (**A**), Podocin (**B**), and Desmin (**C**) in the renal tissues of rats in each group; (**D**) Western blot to detect the protein levels of p-PI3K, PI3K, p-Akt, Akt, p-mTOR, and mTOR in the renal tissues of rats in each group. (**E**–**G**) Quantification of p-PI3K, p-AKT and p-mTOR. n=3. *p < 0.05, **p < 0.01 and ***p < 0.001 vs DKD group.

Next, we explored the detailed mechanism of its protective effect on DKD. We observed that mRNA levels of podocytes markers, such as *nephrin* (Figure 5A) and *podocin* (Figure 5B), were reduced with the increased mRNA level of muscle marker, *desmin* (Figure 5C), which suggested that podocytes were reduced in the kidney of DKD rats and restored by high dose of Qidantang Granule. Moreover, DKD activated PI3K signaling pathway in the kidney, as reflected by the increased protein levels of p-PI3k, p-AKT, and p-mTOR, were significantly restored by Qidantang Granule (Figure 5D–5G). These data indicated that Qidantang Granule ameliorated podocytes and renal injury by inhibiting PI3K/Akt/mTOR signaling pathway in DKD rats.

## DISCUSSION

Our study used STZ injection to induce DKD in rats. DKD rats displayed increased body weight, food intake, water intake and fasting blood glucose, which was consistent with previous publications. Our results revealed that Qidantang Granule dramatically decreased food and water intake but had no effects on body weight and fasting blood glucose levels at the beginning of treatment. However, as treatment continued, body weight and fasting blood glucose levels of DKD rats were gradually decreased after 4 weeks, and further decreased in the following weeks. This result indicated that Qidantang Granule did not act immediately, and it was a long-term accumulation for at least one month. Additionally, this data also demonstrated that beside renal injury, Qidantang Granule might also have protective effect on diabetes.

To further confirm the effect of Qidantang Granule on DKD, we measured renal injury biomarkers both in the kidney tissue and serum, including Scr, BUN, mALB, ACR, right kidney/body weight, urinary β-NAF/urinary creatinine, 24 h urine protein, serum urea nitrogen, serum uric acid and serum creatinine. These biomarkers were at least partially restored by Qidantang Granule treatment. The results indicated that Qidantang Granule effectively attenuated renal injury, which suggested the effectiveness of Qidantang Granule on DKD. Our data also demonstrated that Qidantang Granule also remarkably ameliorated activated inflammation and oxidative stress, which might also facilitate the improvement of Qidantang Granule on diabetic nephropathy.

However, our data were only based on *in vivo* experiments. Although we revealed that PI3K/Akt/mTOR signaling pathway was involved in the process of the protective activities of Qidantang Granule on DKD. But we did not isolate podocytes in the renal tissue to confirm the effect of Qidantang Granule in the development of DKD. Based on the importance of podocytes in the pathology of DKD, it is better to isolate podocytes from renal tissue and treat with different doses of Qidantang Granule to further confirm its effect.

Although preventing podocyte damage was considered as the key to prevent the occurrence of DKD [11], this might not be the only mechanism of the protective role of Qidantang Granule on kidney injury. And we still need to further explore the detailed mechanism of its protective effect of Qidantang Granule on DKD.

## CONCLUSIONS

Our data for the first time revealed that Qidantang Granule effectively ameliorated renal injury, attenuated inflammation and oxidative stress, reduced food intake, water intake, body weight and fasting blood glucose through inhibiting PI3K signaling pathway in STZ-induced DKD rats. Our data provide clear experimental evidence to prove its protective effects on DKD.

## Supplementary Material

Supplementary Figures
