# Supplementary material for: Effects of Qidantang Granule on early stage of diabetic kidney disease in rats
Source: Aging (Albany NY). 2022 Jun 13;14(11):4888–96. doi: 10.18632/aging.204121 (PMC9217703; doi:10.18632/aging.204121)
Supplement: Supplementary Figures [file aging-14-204121-s001.pdf]

SUPPLEMENTARY FIGURES

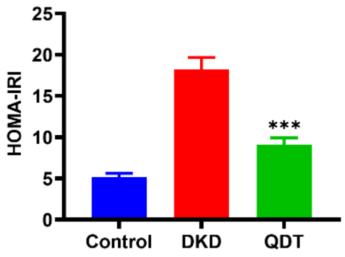

Supplementary Figure 1. Variations of rat HOMA-IRI in rats after Qidantang granule intervention.

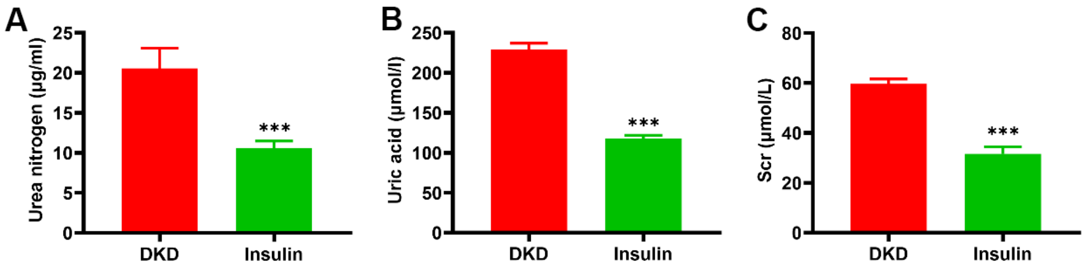

Supplementary Figure 2. Replenishment of insulin reduced kidney damage caused by STZ. (**A**) Serum urea nitrogen. (**B**) Serum uric acid. (**C**) Serum creatinine. Data are expressed as mean SD (n = 6). ***p < 0.001.
